# CHARACTERISTICS OF SUBACUTE STROKE PATIENTS WHO ACHIEVE EARLIER INDEPENDENCE IN REAL-LIFE WALKING PERFORMANCE DURING HOSPITALIZATION

**DOI:** 10.2340/jrm.v57.41993

**Published:** 2025-01-03

**Authors:** Kenji KAWAKAMI, Shigeo TANABE, Daiki KINOSHITA, Ryo KITABATAKE, Hiroo KOSHISAKI, Kenta FUJIMURA, Yoshikiyo KANADA, Hiroaki SAKURAI

**Affiliations:** 1 Faculty of Rehabilitation, School of Health Sciences, Fujita Health University, Toyoake-shi, Aichi; 2 Graduate School of Health Sciences, Fujita Health University, Aichi; 3 Department of Rehabilitation, Kyoto Rehabilitation Hospital, Kyoto-shi, Kyoto; 4 Department of Rehabilitation, Nanto Municipal Hospital, Toyama, Japan

**Keywords:** cerebrovascular disorders, hemiplegia, inpatient, walking

## Abstract

**Objective:**

To identify factors associated with earlier independence in “real-life walking” during hospitalization in subacute stroke patients.

**Design:**

Retrospective cohort study.

**Subjects/Patients:**

Two hundred and six hemiplegic patients.

**Methods:**

Functional Independence Measure (FIM) walking items were measured biweekly from admission to discharge. Patients were grouped by achieving independent “real-life walking” (FIM-walking score ≥6). Time to independence, stratified by age, FIM motor score (FIM-M), FIM cognitive score (FIM-C), and Functional Ambulation Categories (FAC) scores were compared using Kaplan– Meier plots and log-rank tests. Hazard ratios were calculated via multivariable Cox proportional hazard models.

**Results:**

The median time to independence was 4 weeks, with significant differences (*p* < 0.05) by age, FIM-M, FIM-C, and FAC stratification. Age ≤64 years (hazard ratio 1.92, 95% confidence interval 1.21–3.06), FIM-C ≥25 (hazard ratio 2.42, 95% confidence interval 1.52–3.86), and FAC ≥3 (hazard ratio 1.98, 95% confidence interval 1.22–3.21) significantly affected earlier walking independence (all *p* < 0.01). Impeding factors were FIM-M ≤38 (hazard ratio 0.23, 95% confidence interval 0.13–0.40; *p* < 0.01) and FAC = 0 (hazard ratio 0.184, 95% confidence interval 0.06–0.62; *p* < 0.01).

**Conclusion:**

Early improvement in “real-life walking” was associated with younger age, greater cognitive function, and greater “test-setting walking” ability on admission. Low activities of daily living independence and “test-setting walking” ability hindered early progress.

## LAY ABSTRACT

This study focused on identifying factors influencing early walking independence in patients with stroke during hospitalization. While most research on walking outcomes in patients with stroke emphasizes “test-setting walking” in controlled environments, this study examined “real-life walking” in daily settings, which is less frequently studied and typically only assessed at a single time point. Researchers followed 206 patients with stroke in a rehabilitation ward and regularly assessed their real-life walking ability. The study revealed that most patients achieved walking independence after approximately 4 weeks. Factors that promoted early independence included being younger than 64 years old, having better cognitive function, and having better walking ability on admission. Conversely, low independence in activities of daily living and poor walking skills at the start of rehabilitation slowed progress. These findings can help healthcare teams develop more effective rehabilitation plans for patients with stroke.

Gait disturbance is a significant poststroke issue that impacts activities of daily living (ADL) and quality of life (1). Accurate prediction of independent walking facilitates rehabilitation planning and setting goals (2). Research has indicated that age (3, 4), lower extremity muscle strength on the paralysed side (2), cognitive function (4), and ADL level (5) at the time of stroke influence walking ability. Kennedy et al. (6) studied the number of days until walking in a test situation 3 months after stroke onset and reported a median of 6 days. They reported that older age, diabetes, severe stroke, haemorrhagic stroke, and right hemisphere stroke were associated with delayed improvement in walking independence. These studies focused on “test-setting walking” ability in controlled environments, such as the Functional Ambulation Categories (FAC) (7) and 10-m walking speed assessments (8).

However, identifying the characteristics of patients who achieve early improvement in “real-life walking” during hospitalization can assist in setting rehabilitation goals and planning in-hospital walking programmes. Thus, research on predicting “real-life walking” performance outcomes is needed (9). The Functional Independence Measure (FIM) (10), an ADL-evaluation method in real-life settings, includes 18 subitems, of which 13 are motor and 5 are cognitive items. The “FIM-walking” item practically evaluates functional performance during hospitalization, including daytime and nighttime activities, in contrast with “walking ability in test environments” (10).

To date, few reports have predicted “real-life walking” independence during hospitalization. Makizako et al. (11) reported that the Berg Balance Scale (12) score on admission predicted walking independence in real-life situations 3 months after admission in patients with stroke at a convalescent rehabilitation hospital. Ishiwatari et al. (13) reported that trunk function on admission could predict walking independence in real-life situations on discharge in patients with stroke at an acute care hospital. However, these studies focused on assessments at a single time point rather than identifying the characteristics of patients who achieve early independence.

Although these studies provided valuable insights into predictors of discharge outcomes, they primarily assessed walking ability at a single time point, such as discharge. They did not investigate factors associated with rapid recovery of walking ability during hospitalization. Therefore, our study could fill a gap in the literature by examining factors associated with early independence in “real-life walking” performance during hospital stays.

In a recent systematic review, Meyer et al. (14) identified factors impacting discharge FIM total scores, such as admission functional level, National Institute of Health Stroke Scale (15), dysphasia, impulsivity, neglect, previous stroke, and age. However, they did not predict individual functional components such as walking (14). Although Chevalley et al. (16) also reported in their systematic review that admission FIM motor scores above 26 indicated a greater likelihood of returning home, they did not address the rate of functional improvement during hospitalization. This highlights the need for more specific subscore analyses and time-sensitive assessments.

This study aimed to identify factors influencing early walking independence in real-life situations by examining the FIM-walking score in patients with subacute stroke in a convalescent rehabilitation ward.

## METHODS

### Participants

**Fig. 1:**
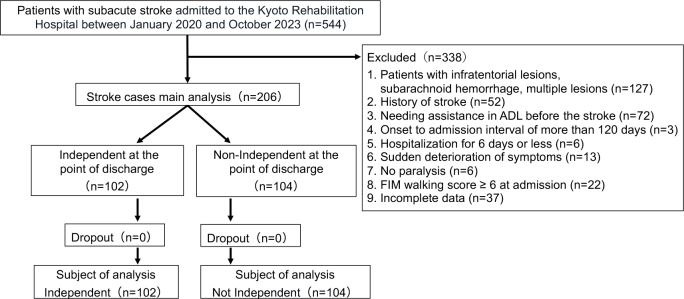
Flow diagram of patient enrolment and study completion. Among the 544 patients with hemiplegia admitted to the Kyoto Rehabilitation Hospital between January 2020 and October 2023, 206 were included in the analysis and 338 were excluded on the basis of the exclusion criteria. The number of patients included in the study was 102 and 104 for the Independent and Non-Independent groups, respectively. FIM: Functional Independence Measure.

We conducted a retrospective cohort study of patients with stroke admitted to a convalescent hospital. The inclusion criterion was patients with subacute stroke admitted to Kyoto Rehabilitation Hospital between January 2020 and October 2023. The exclusion criteria were patients with infratentorial lesions, subarachnoid haemorrhage, multiple lesions, a history of stroke, needing assistance in ADL before the stroke, an onset-to-admission interval of more than 120 days, hospitalization for 6 days or less, sudden deterioration of symptoms, no paralysis, FIM-walking score ≥6 on admission, and incomplete data. Among the 544 patients with hemiplegia admitted during the same period, 206 were included in the analysis after 338 who met the exclusion criteria were excluded (Fig. 1). All patients underwent physical and occupational therapy and speech therapy, if needed, for 2.5–3 h daily, 7 days a week, from admission to discharge, in accordance with the Full-time Integrated Treatment (FIT) programme for standardizing training content and intensity (17). This programme aimed to facilitate a standardized approach among therapists by setting exercise difficulty levels on the basis of motor learning principles, focusing on ADL and walking training. Additionally, the FIT programme helped minimize variability in therapists’ levels of expertise or treatment approaches through a team-based structure, utilizing a dual therapist system with designated primary and secondary therapist roles (17). In addition, the rehabilitation plan, goal setting, and length of stay during hospitalization were determined at regular conferences held by the attending physician, nurses, physical therapists, occupational therapists, speech therapists, medical social workers, and national registered dietitians.

This study was approved by the Ethics Committee of Fujita Health University (approval number: HM22-504; August 10, 2021) and registered in the University Hospital Medical Information Network-Clinical Trials Registry (UMIN-CTR UMIN 000053209). Since this was a retrospective observational analysis using only existing data, no written or verbal consent was required or obtained from the patients. Study details were disclosed to the patients so that they could decline their data’s use for research (opt-out).

### Measurements

Upon admission, data on patient age, sex, stroke type, paralysed side, number of days from onset to hospital admission, number of days from onset to hospital discharge, total lower-extremity motor function score of the Stroke Impairment Assessment Set (SIAS LE) (18), total FIM-motor item score (FIM-M) (10), FIM-walking score (a subitem of FIM-M) (10), total FIM-cognitive item score (FIM-C) (10), and FAC score (7) were recorded. Additionally, the primary endpoint variable, FIM-walking, was assessed every 2 weeks from admission to discharge. In our study, we defined “real-life walking” as the performance of practical walking in a structured hospital ward. Specifically, patients were evaluated when they rose from their hospital beds, opened doors to their rooms, navigated around other patients who might be walking in the hallway, and proceeded to their designated destinations, such as the restroom or dining area. Additionally, this assessment included conditions during daytime and nighttime and immediately upon awakening. The FIM (10) is an ADL evaluation method consisting of 18 evaluation items, 13 of which address motor and 5 of which address cognitive issues. Each item is rated on a 7-point Likert scale from 1 to 7, depending on the degree of assistance needed. The FIM-walking subitem is evaluated under conditions of actual walking performance in real-life situations. Independence in this study was defined as an FIM-walking score of 6 or higher (6 = modified independence, 7 = complete independence). Complete independence (score of 7) means that the individual can safely walk at least 150 ft (50 m) without assistive devices. Modified independence (score of 6) means that the individual can walk at least 150 ft (50 m) without supervision but with the support of an orthosis, cane, or walkerette. Specifically, a FIM-walking score of 6 or higher indicates that the patient can independently ambulate in the actual ward setting without the need for assistance or supervision. FIM scores of 1–5 are determined by the level of physical assistance required for walking and the presence of supervision. The reliability and validity of this scale have been confirmed in patients with stroke (19). In this study, FIM assessments and recordings were conducted by physical and occupational therapists who received training and certification in FIM use.

### Data analysis and statistical processing

Patients scoring 6 or higher at the point of discharge were allocated into the Independent group, whereas those scoring 5 or lower at the point of discharge were included in the Non-Independent group. The number of patients for analysis was set at 102 in the Independent group and 104 in the Non-Independent group (Fig. 1). Patient information on admission was compared between the Independent and Non-Independent groups using the Mann–Whitney *U* test for ordinal variables and the Pearson χ^2^ test for nominal variables. We then performed a survival-time analysis using improvement to a FIM-walking score of 6 or higher as the event during hospitalization, referencing previous studies (6), Kaplan–Meier plots, and log-rank tests. Survival-time curves were compared and stratified by age, FIM-M score, FIM-C score, and FAC score on admission. The number of weeks from admission until the FIM-walking score improved to 6 or higher was assigned as the time variable. Patients discharged without a FIM-walking score of 6 or higher during hospitalization were censored at the number of weeks from admission to discharge.

Additionally, the probability of achieving a FIM-walking score of 6 or higher during hospitalization was calculated using a multivariate Cox proportional hazards model. Age (3, 4), FIM-M score (20), FIM-C score (21), which have previously been reported to influence walking ability, and FAC score on admission were included as explanatory variables to estimate hazard ratios (HRs). The forward selection (likelihood ratio) method was used for variable extraction. To account for bias in the distribution of scores and because high or low scores for each variable may have reciprocal effects on the results, the variables were stratified as follows on the basis of previous reports. Age was stratified into 64 years or younger (low age), 65–80 years (middle age), and 81 years or older (high age) (6); FIM-M score was stratified into 13–38 points (low ADL), 39–64 points (middle ADL), and 65–91 points (high ADL) (22); and FIM-C score was stratified into 5–14 points (low cognition), 15–24 points (medium cognition), and 25–35 points (high cognition) (22). FAC scores were classified as 0, 1, 2, or 3 or more points. These classifications were used in the model.

SPSS software (version 16.0 for Mac; IBM Japan Ltd, Tokyo, Japan) was used for statistical analyses. The significance level was set at 5%.

## RESULTS

### Patient characteristics

None of the patients declined participation or passed away during hospitalization. Table I shows patient information on admission. The Independent group was significantly younger (mean [standard deviation (SD)]: Independent group, 71.12 [13.67] years; Non-Independent group, 77.64 [9.00] years; *p* < 0.001), had a shorter time from onset to admission (mean [SD]: Independent group, 24.72 [13.73] days; Non-Independent group, 29.87 [15.07] days; *p* = 0.003), had a shorter time from onset to discharge (mean [SD]: Independent group, 105.66 [39.35] days; Non-Independent group, 140.95 [48.80] days; *p* < 0.001), and had higher SIAS LE scores (median [interquartile range (IQR)]: Independent group 12 [9–14]; Non-Independent group, 6 [1–12]; *p* < 0.001) than did the Non-Independent group on admission. Compared with the Non-Independent group, the Independent group had greater FIM-M (median [IQR]: Independent group, 50.5 [38–60]; Non-Independent group, 19 [13–30]; *p* < 0.001), FIM-walking (median [IQR]: Independent group, 1 [1–4]; Non-Independent group, 1 [1–1], *p* < 0.001), FIM-C (median [IQR]: Independent group, 27 [21–31]; Non-Independent group, 16.5 [10–21]; *p* < 0.001), and FAC (median [IQR]: Independent group, 2 [1–3]; Non-Independent group, 1 [0–2]; *p* < 0.001).

**Table I:** Patient characteristics on admission

Variable	Total [*n* = 206]	Independent [*n* = 102]	Non-Independent [*n* = 104]	*p*-value
Age in years, mean ± SD [median]	74.41 ± 11.98 [76]	71.12 ± 13.67 [72.5]	77.64 ± 9.00 [78.5]	< 0.001^a^
Sex (male/female)	105/115	46/56	45/59	0.566^b^
Stroke type (haemorrhage/infarction)	87/119	41/61	46/58	0.558^b^
Paralysed side (right/left)	105/101	49/53	56/48	0.843^b^
Days from onset to hospital admission, mean ± SD [median]	27.32 ± 14.61 [23]	24.72 ± 13.73 [21]	29.87 ± 15.07 [25.5]	0.003^a^
Days from onset to hospital discharge, mean ± SD [median]	123.48 ± 47.67 [122]	105.66 ± 39.35 [99.5]	140.95 ± 48.80 [142]	< 0.001^a^
SIAS LE, median [IQR]	11 [4–12]	12 [9–14]	6 [1–12]	< 0.001^a^
FIM-M, median [IQR]	33.5 [17–52]	50.5 [38–60]	19 [13–30]	< 0.001^a^
FIM-walking, median [IQR]	1 [1–1]	1 [1–4]	1 [1–1]	< 0.001^a^
FIM-C, median [IQR]	21 [14–28]	27 [21–31]	16.5 [10–21]	< 0.001^a^
FAC, median [IQR]	1 [1–3]	2 [1–3]	1 [0–2]	< 0.001^a^

a Mann‒Whitney *U* test,

b Pearson χ^2^ test.

SD: standard deviation; IQR: interquartile range; SIAS LE: Stroke Impairment Assessment Set Lower Extremities; FIM-M: Functional Independence Measure Motor Items; FIM-walking: Functional Independence Measure walking; FIM-C: Functional Independence Measure Cognitive Items; FAC: Functional Ambulation Categories.

### Time to achieving a FIM-walking score of 6 or higher and its relationship with each variable

**Fig. 2:**
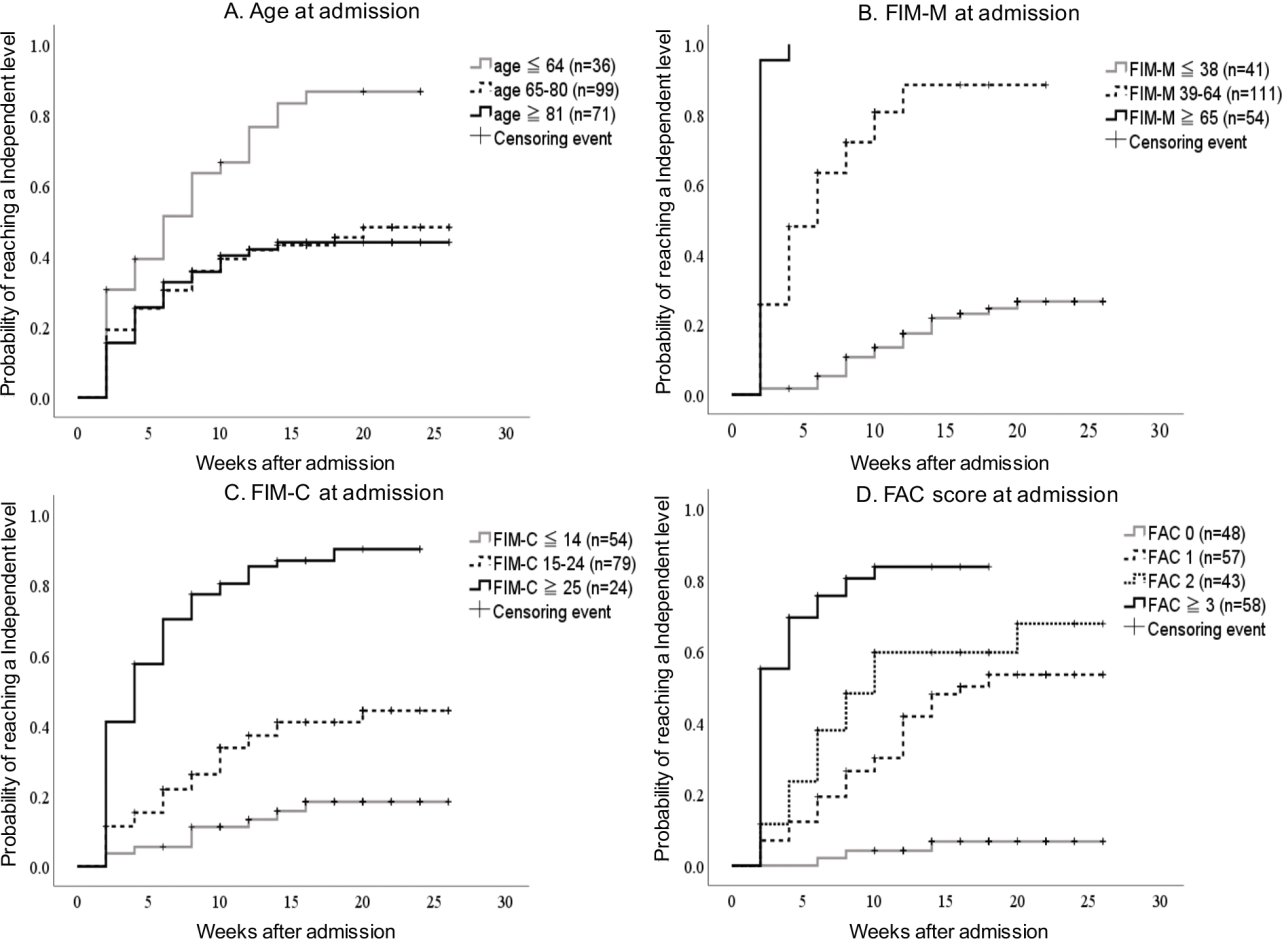
Kaplan–Meier curve analysis for reaching an independent walking level according to (A) age, (B) FIM-M, (C) FIM-C, and (D) FAC scores. The values are not adjusted for age or FIM-M, FIM-C, or FAC scores. Significant differences were observed in the probability of reaching the independent walking level in analyses stratified by age on admission (log-rank test, *p* < 0.001), FIM-M on admission (log-rank test, *p* < 0.001), FIM-C on admission (log-rank test, *p* < 0.001), and FAC score on admission (log-rank test, *p* < 0.001). FIM-M: Functional Independence Measure Motor Items; FIM-C: Functional Independence Measure Cognitive Items; FAC: Functional Ambulation Categories.

The median time from admission to achieving an FIM-walking score of 6 or higher was 4 weeks (IQR: 2–8 weeks). From admission to discharge, 102 patients (49.5%) could walk independently in real-life situations. Fig. 2 shows the relationships between age, FIM-M, FIM-C, and FAC on admission and the time to achieving a FIM-walking score of 6 or higher using Kaplan–Meier curves without adjustment for covariates. The log-rank test was significant (P < 0.05) in each analysis, indicating that the time to achieving an FIM-walking score of 6 or higher and the percentage of patients who achieved this goal differed based on age, ADL, cognitive function, and “test-setting walking” ability on admission (Fig. 2).

### Factors associated with the time to achieving a FIM-walking score of 6 or higher according to multivariate Cox proportional hazards analysis

Multivariate Cox proportional hazards analysis identified independent factors positively associated with achieving a FIM-walking score of 6 or higher early during hospitalization: age ≤64 years (HR 1.92, 95% confidence interval [CI] 1.21–3.06, *p* < 0.01), FIM-C ≥25 (HR 2.42, 95% CI 1.52–3.86, *p* < 0.01), and FAC ≥3 (HR 1.98, 95% CI 1.22–3.21, *p* < 0.01) on admission (Table II). On the other hand, FIM-M ≤ 38 (HR 0.23, 95% CI 0.13–0.40, *p* < 0.01) and FAC = 0 (HR 0.18, 95% CI 0.06–0.62, *p* < 0.01) on admission were identified as factors that inhibited early achievement of walking independence.

**Table II:** Multivariate Cox proportional hazards analysis of achieving a FIM-walking score of 6 or higher

Factor	B	Hazard ratio	*p*-value	95.0% CI of hazard ratio	
				Lower	Upper
Age ≤64	0.653	1.922	0.006	1.208	3.058
FIM-M ≤38	–1.471	0.230	0.000	0.133	0.398
FIM-C ≥25	0.884	2.421	0.000	1.518	3.863
FAC ≥3	0.681	1.976	0.006	1.217	3.208
FAC = 0	–1.692	0.184	0.006	0.055	0.615

FAC: Functional Ambulation Categories; FIM-M: Functional Independence Measure Motor Items; FIM-C: Functional Independence Measure Cognitive Item.

## DISCUSSION

In this study, factors associated with early independence in “real-life walking” were examined in patients with subacute stroke admitted to a convalescent rehabilitation ward. By following the course of patients’ “real-life walking” performance, we found that the time required to achieve walking independence in real-life situations and the rate of achievement varied depending on age, ADL performance status, cognitive function, and “test-setting walking” ability on admission. Multivariate Cox proportional hazards analysis adjusted for these variables revealed that younger age, greater cognitive function, and greater “test-setting walking” ability on admission promoted early walking independence in real-life situations. We also showed that low ADL ability and low “test-setting walking” ability on admission inhibited early walking independence in real-life situations. Accordingly, this study revealed the course of walking performance in real-life situations and examined the factors necessary for early independence in walking in the same conditions, which has not been reported previously.

In a previous report on the progression of “test-setting walking” ability, the median time from stroke onset to “walking 50 m unassisted” was 6 days (IQR: 2–63 days), which was achieved by 75% of patients (6). Despite differences in patient characteristics (e.g., motor paralysis), this study revealed that “real-life walking” performance reached an independent level after a median of 4 weeks following admission to a convalescent rehabilitation ward. Given that the median time from onset to hospitalization was 21 days, the total duration was approximately 7 weeks, indicating a longer period needed to recover walking ability. A previous study reported that 75% of patients achieved independence in “test-setting walking” within 3 months post-onset. In contrast, in this study, only 49.5% achieved independence in “real-life walking” by the time of discharge (median, 122 days post-onset). Thus, in terms of “real-life walking”, more time is required to achieve independence, and a lower percentage of independence is achieved than reflected by “test-setting walking” ability. These findings suggest that independence in “test-setting walking” does not guarantee independence in real-life walking and underscores the need to focus on “real-life walking” performance in future research.

A younger age significantly influenced early independence in “real-life walking” performance. Research has indicated that older age negatively impacts “test-setting walking” at 6 months poststroke (23), aligning with our findings. Ageing leads to a decline in maximum limb muscle strength, oxygen consumption, sensory functions, and reduced daily steps and walking speed (24). Additionally, patients over 65 years of age who were hospitalized for acute illness were reported to be more susceptible to functional decline and decreased ADL (25). Thus, younger patients are more likely to achieve independence in “real-life walking” earlier in their recovery process than older patients.

High cognitive function on admission is suggested to influence early independence in “real-life walking”. The FIM-C, used as a cognitive function assessment scale in this study, has shown a significant positive correlation with the Mini-Mental State Examination (MMSE) (26) and the Loewenstein Occupational Therapy Cognitive Assessment (27), making it suitable for assessing cognitive function in patients with stroke (28). Cognitive function in the acute phase of stroke is a known predictor of “test-setting walking” ability (4), and this study confirmed its influence on early independence in real-life walking. Oros et al. (29) analysed the correlation between MMSE scores and ADL in poststroke patients and reported that those with cognitive impairment were more dependent on caregivers and experienced greater decreases in ADL (29, 30). Therefore, patients with high cognitive function on admission are expected to be less dependent on caregivers, resulting in greater improvements in ADL, including walking, which may influence early achievement of walking independence in real-life situations.

Upon admission, patients with higher FAC scores, indicating better “test-setting walking” ability, achieved walking independence earlier in real-life situations. Previous studies have shown that reduced walking ability poststroke increases fall risk (31). The FAC is positively correlated with the Rivermead Mobility Index, which is also strongly associated with falls (7), suggesting that higher scores on these scales correlate with a lower fall risk (32). Thus, a greater ability to walk early in hospitalization reduces the risk of falls and accelerates independent walking in real-life situations. Faria-Fortini et al. (1) explored the relationship between walking speed and participation in ADL and reported a positive correlation with activities such as getting in and out of bed, using baths and toilets, and doing housework. Given the strong positive correlation between FAC and walking speed (33), it is likely that patients with higher FAC scores on admission had greater ADL participation, enabling earlier independent walking in real-life situations.

In contrast, low ADL and low “test-setting walking” abilities on admission were identified as factors that inhibit early walking independence in real-life situations. Some patients may not have achieved walking independence during their hospitalization period, possibly due to the high severity and extensive impact of the stroke or a potential decline in the function of the nonaffected hemisphere (34).

In this study, we identified the characteristics of patients who are likely to achieve early independence in “real-life walking” and those who are likely to have difficulty achieving independence. Therefore, predicting when patients will likely achieve real-life walking independence upon admission to the rehabilitation ward could be clinically important for developing specific discharge plans. Sharing these findings with physicians, nurses, and therapists at multidisciplinary conferences is expected to help create more effective rehabilitation programmes.

### Limitations

A limitation of this study is that although the FIM score was used to measure “real-life walking” performance during hospitalization, how accurately this measurement could reflect patients’ walking ability in more complex and unpredictable everyday situations outside the hospital environment remains unclear. Community ambulation poststroke has been reported to be affected by more complex factors than walking in a structured environment such as a hospital ward (35). Additionally, this study included patients with haemorrhagic stroke as well as severe stroke, who are expected to take longer to achieve independence (6), but the study was not validated after censoring at the point of discharge. Future research should include situations outside ward life and follow-up assessments after discharge. Furthermore, the content and intensity of rehabilitation may affect the outcomes of walking performance (36–38). However, owing to the difficulty in collecting information on the specific content and intensity of rehabilitation during hospitalization, this remains a challenge for future studies. Different countries and cultures may have different rehabilitation outcomes (39). The study highlights that, compared with high-resource settings, clinical practice guidelines for stroke rehabilitation are often underutilized in low- and middle-income countries owing to barriers such as limited resources, which could affect recovery outcomes (40). Our study represents a step towards understanding the specific patient population in Japan, but further validation is needed in diverse rehabilitation settings. Finally, the possibility of selection bias cannot be ruled out because most severely affected patients might not have participated in the study due to excluding patients with a history of stroke or multiple lesions. Owing to the retrospective design of this study, selective outcome reporting bias cannot be eliminated, and measurement accuracy may be limited. Future research should aim to validate these results through prospective studies.

### Conclusions

Among patients with subacute stroke who were admitted to a convalescent rehabilitation ward, those who were younger, had greater cognitive function, and exhibited greater “test-setting walking” ability on admission were likely to experience earlier improvements in “real-life walking” performance. Additionally, patients with lower ADL independence and reduced “test-setting walking” ability on admission were less likely to show improvement. This information could facilitate the development of more efficient rehabilitation plans.

## References

[1] Faria-Fortini I, Polese JC, Faria CDCM, Teixeira-Salmela LF. Associations between walking speed and participation, according to walking status in individuals with chronic stroke. NeuroRehabilitation 2019; 45: 341–348. 10.3233/NRE-192805 31796694

[2] Smith MC, Barber PA, Stinear CM. The TWIST algorithm predicts time to walking independently after stroke. Neurorehabil Neural Repair 2017; 31: 955–964. 10.1177/1545968317736820 29090654

[3] Aaslund MK, Moe-Nilssen R, Gjelsvik BB, Bogen B, Næss H, Hofstad H, et al. A longitudinal study investigating how stroke severity, disability, and physical function the first week post-stroke are associated with walking speed six months post-stroke. Physiother Theor Pract 2017; 33: 932–942. 10.1080/09593985.2017.1360424 28816573

[4] Park J, Lee SU, Jung SH. Prediction of post-stroke functional mobility from the initial assessment of cognitive function. NeuroRehabilitation 2017; 41: 169–177. 10.3233/NRE-171469 28505995

[5] Paolucci S, Bragoni M, Coiro P, De Angelis D, Fusco FR, Morelli D, et al. Quantification of the probability of reaching mobility independence at discharge from a rehabilitation hospital in nonwalking early ischemic stroke patients: a multivariate study. Cerebrovasc Dis 2008; 26: 16–22. 10.1159/000135648 18511867

[6] Kennedy C, Bernhardt J, Churilov L, Collier JM, Ellery F, Rethnam V, et al. Factors associated with time to independent walking recovery post-stroke. J Neurol Neurosurg Psychiatry 2021; 92: 702–708. 10.1136/jnnp-2020-325125 33737383

[7] Holden MK, Gill KM, Magliozzi MR, Nathan J, Piehl-Baker L. Clinical gait assessment in the neurologically impaired: reliability and meaningfulness. Phys Ther 1984; 64: 35–40. 10.1093/ptj/64.1.35 6691052

[8] Collen FM, Wade DT, Bradshaw CM. Mobility after stroke: reliability of measures of impairment and disability. Int Disabil Stud 1990; 12: 6–9. 10.3109/03790799009166594 2211468

[9] Moore SA, Boyne P, Fulk G, Verheyden G, Fini NA. Walk the talk: current evidence for walking recovery after stroke, future pathways and a mission for research and clinical practice. Stroke 2022; 53: 3494–3505. 10.1161/STROKEAHA.122.038956 36069185 PMC9613533

[10] Data management service of the Uniform Data System for Medical Rehabilitation and the Center for Functional Assessment Research. Guide for use of the uniform data set for medical rehabilitation (ver.3.0). New York: State University of New York at Buffalo; 1990.

[11] Makizako H, Kabe N, Takano A, Isobe K. Use of the Berg Balance Scale to predict independent gait after stroke: a study of an inpatient population in Japan. PM R 2015; 7: 392–399. 10.1016/j.pmrj.2015.01.009 25633633

[12] Blum L, Korner-Bitensky N. Usefulness of the Berg Balance Scale in stroke rehabilitation: a systematic review. Phys Ther 2008; 88: 559–566. 10.2522/ptj.20070205 18292215

[13] Ishiwatari M, Tani M, Isayama R, Honaga K, Hayakawa M, Takakura T, et al. Prediction of gait independence using the Trunk Impairment Scale in patients with acute stroke. Ther Adv Neurol Disord 2022; 15: 17562864221140180. 10.1177/17562864221140180 36506941 PMC9730005

[14] Meyer MJ, Pereira S, McClure A, Teasell R, Thind A, Koval J, et al. Systematic review of studies reporting multivariable models to predict functional outcomes after post-stroke inpatient rehabilitation. Disabil Rehabil 2015; 37: 1316–1323. 10.3109/09638288.2014.963706 25250807

[15] Brott T, Adams HP Jr, Olinger CP, Marler JR, Barsan WG, Biller J, et al. Measurements of acute cerebral infarction: a clinical examination scale. Stroke 1989; 20: 864–870. 10.1161/01.STR.20.7.864 2749846

[16] Chevalley O, Truijen S, Opsommer E, Saeys W. Physical functioning factors predicting a return home after stroke rehabilitation: a systematic review and meta-analysis. Clin Rehabil 2023; 37: 1698–1716. 10.1177/02692155231185446 37424501 PMC10580673

[17] Sonoda S, Saitoh E, Nagai S, Kawakita M, Kanada Y. Full-time integrated treatment program, a new system for stroke rehabilitation in Japan: comparison with conventional rehabilitation. Am J Phys Med Rehabil 2004; 83: 88–93. 10.1097/01.PHM.0000107481.69424.E1 14758294

[18] Chino N, Sonoda S, Domen K, Saitoh E, Kimura A. Stroke impairment assessment set (SIAS): a new evaluation instrument for stroke patients. Jpn J Rehabil Med 1994; 31: 119–125. 10.2490/jjrm1963.31.119

[19] Ottenbacher KJ, Hsu Y, Granger CV, Fiedler RC. The reliability of the functional independence measure: a quantitative review. Arch Phys Med Rehabil 1996; 77: 1226–1232. 10.1016/S0003-9993(96)90184-7 8976303

[20] Petrilli S, Durufle A, Nicolas B, Pinel JF, Kerdoncuff V, Gallien P. Prognostic factors in the recovery of the ability to walk after stroke. J Stroke Cerebrovasc Dis 2002; 11: 330–335. 10.1053/jscd.2002.130124 17903895

[21] Itoh S, Ogino T, Kawakami K, Miyake K, Iyoda H, Imaizumi H, et al. Gait evaluation in stroke hemiplegic patients using short physical performance battery. J Stroke Cerebrovasc Dis 2022; 31: 106704. 10.1016/j.jstrokecerebrovasdis.2022.106704 36037677

[22] Imada Y, Tokunaga M, Fukunaga K, Sannomiya K, Inoue R, Hamasaki H, et al. Relationship between cognitive FIM score and motor FIM gain in patients with stroke in a Kaifukuki rehabilitation ward. Jpn J Compr Rehabil Sci 2014; 5: 12–18. 10.11336/jjcrs.5.12

[23] Rodrigues NAG, da Silva SLA, Nascimento LR, de Paula Magalhães J, Sant’Anna RV, de Morais Faria CDC, et al. R3-walk and R6-walk, simple clinical equations to accurately predict independent walking at 3 and 6 months after stroke: a prospective, cohort study. Arch Phys Med Rehabil 2024; 105: 1116–1123. 10.1016/j.apmr.2024.01.013 38281578

[24] Grimmer M, Riener R, Walsh CJ, Seyfarth A. Mobility related physical and functional losses due to aging and disease: a motivation for lower limb exoskeletons. J Neuroeng Rehabil 2019; 16: 2. 10.1186/s12984-018-0458-8 30606194 PMC6318939

[25] Geyskens L, Jeuris A, Deschodt M, Van Grootven B, Gielen E, Flamaing J. Patient-related risk factors for in-hospital functional decline in older adults: a systematic review and meta-analysis. Age Ageing 2022; 51: afac007. 10.1093/ageing/afac007 35165688

[26] Folstein MF, Folstein SE, McHugh PR. “Mini-mental state”: a practical method for grading the cognitive state of patients for the clinician. J Psychiatr Res 1975; 12: 189–198. 10.1016/0022-3956(75)90026-6 1202204

[27] Askenasy JJ, Rahmani L. Neuropsycho-social rehabilitation of head injury. Am J Phys Med 1987; 66: 315–327. 10.1097/00002060-198812000-00001 3124629

[28] Zwecker M, Levenkrohn S, Fleisig Y, Zeilig G, Ohry A, Adunsky A. Mini-Mental State Examination, cognitive FIM instrument, and the Loewenstein occupational therapy Cognitive Assessment: relation to functional outcome of stroke patients. Arch Phys Med Rehabil 2002; 83: 342–345. 10.1053/apmr.2002.29641 11887114

[29] Oros RI, Popescu CA, Iova CA, Mihancea P, Iova SO. The impact of cognitive impairment after stroke on activities of daily living. Human Vet Med 2016; 8: 41–44.

[30] Suda S, Nishimura T, Ishiwata A, Muraga K, Aoki J, Kanamaru T, et al. Early cognitive impairment after minor stroke: associated factors and functional outcome. J Stroke Cerebrovasc Dis 2020; 29: 104749. 10.1016/j.jstrokecerebrovasdis.2020.104749 32178931

[31] Persson CU, Hansson PO, Sunnerhagen KS. Clinical tests performed in acute stroke identify the risk of falling during the first year: postural stroke study in Gothenburg (POSTGOT). J Rehabil Med 2011; 43: 348–353. 10.2340/16501977-0677 21267528

[32] Kose N, Cuvalci S, Ekici G, Otman AS, Karakaya MG. The risk factors of fall and their correlation with balance, depression, cognitive impairment and mobility skills in elderly nursing home residents. Saudi Med J 2005; 26: 978–981. 15983687

[33] Mehrholz J, Wagner K, Rutte K, Meissner D, Pohl M. Predictive validity and responsiveness of the functional ambulation category in hemiparetic patients after stroke. Arch Phys Med Rehabil 2007; 88: 1314–1319. 10.1016/j.apmr.2007.06.764 17908575

[34] Wandel A, Jørgensen HS, Nakayama H, Raaschou HO, Olsen TS. Prediction of walking function in stroke patients with initial lower extremity paralysis: the Copenhagen Stroke Study. Arch Phys Med Rehabil 2000; 81: 736–738. 10.1016/S0003-9993(00)90102-3 10857515

[35] Bansal K, Morgan-Daniel J, Chatterjee SA, Rose DK. Factors affecting community ambulation post-stroke: a mapping review protocol. F1000Res 2024; 13: 166. 10.12688/f1000research.144582.2 39220386 PMC11364963

[36] Kawakami K, Miyasaka H, Hioki Y, Furumoto A, Sonoda S. Gait training with a safety suspension device accelerates the achievement of supervision level walking in subacute stroke: a randomized controlled trial. Int J Rehabil Res 2024; 47: 75–80. 10.1097/MRR.0000000000000625 38595089

[37] Kawakami K, Tanino G, Tomida K, Kato Y, Watanabe M, Okuyama Y, et al. Influence of increased amount of exercise on improvements in walking ability of convalescent patients with post-stroke hemiplegia. J Phys Ther Sci 2016; 28: 602–606. 10.1589/jpts.28.602 27065551 PMC4793018

[38] Tapp A, Griswold D, Dray D, Landgraff N, Learman K. High-intensity locomotor training during inpatient rehabilitation improves the discharge ambulation function of patients with stroke: a systematic review with meta-analysis. Top Stroke Rehabil 2024; 31: 431–445. 10.1080/10749357.2024.2304960 38285888

[39] Mulder M, Nijland RHM, Vloothuis JDM, van den Berg M, Crotty M, Kwakkel G, et al. Comparing two identically protocolized, multicentre, randomized controlled trials on caregiver-mediated exercises poststroke: any differences across countries? PLoS One 2022; 25: e0263013. 10.1371/journal.pone.0263013 PMC8789096 35077507

[40] Hombali A, Mahmood A, Gandhi DBC, Kamalakannan S, Chawla NS, D’souza J, et al. Clinical practice guidelines (CPGs) for stroke rehabilitation from low- and middle-income countries (LMICs): protocol for systematic review. PLoS One 2023; 18: e0293733. 10.1371/journal.pone.0293733 37943755 PMC10635447

